# Anatomical pathways and functional implications of the rodent auditory system-basal ganglia interconnectivity

**DOI:** 10.3389/fnbeh.2025.1645035

**Published:** 2025-08-22

**Authors:** Ryohei Tomioka, Makoto Takemoto, Wen-Jie Song

**Affiliations:** ^1^Department of Sensory and Cognitive Physiology, Graduate School of Medical Sciences, Kumamoto University, Kumamoto, Japan; ^2^Center for Metabolic Regulation of Healthy Aging, Faculty of Life Sciences, Kumamoto University, Kumamoto, Japan

**Keywords:** auditory cortex, auditory thalamus, lemniscal, non-lemniscal, tail of striatum, tri-laminar tail of striatum, posterior globus pallidus, cuneiform nucleus

## Abstract

Sound influences motor functions and sound perception is conversely modulated by locomotion. Accumulating evidence supports an interconnection between the auditory system and the basal ganglia (BG), which has functional implications on the interaction between the two systems. Substantial evidence now supports auditory cortex and auditory thalamus inputs to the tri-laminar region of the tail of the striatum (tTS) in rodents. Thalamic input modulates the response gain of striatal neurons, whereas cortical input shapes their frequency tuning. Only recently has our understanding of BG projections to the auditory system advanced. GABAergic neurons in the tTS, which receive input from the auditory cortex, project to the posterior globus pallidus external segment (GPe). Posterior GPe, in turn, sends strong GABAergic projections to the non-lemniscal auditory thalamus (NLAT) and moderate projections to the cuneiform nucleus (CnF). The BG and auditory system are thus interconnected at multiple levels, forming a loop circuit in which the auditory system projects to the striatum and receives BG output via the NLAT. This circuit may mediate BG influence on auditory processing; however, the absence of motor cortex input to the tTS raises questions about its role in movement-related modulation of auditory responses. Given that the NLAT serves as a neural substrate for sound-cued aversive associative learning, BG output to the NLAT may influence learning processes. The pathway connecting the auditory system and CnF via the BG may underlie rhythmic entrainment in healthy individuals and therapeutic effects of rhythmic cues on gait in Parkinson’s disease.

## 1 Introduction

The basal ganglia (BG) are involved in movement control, action selection, habit learning, reward processing, and motivational regulation (Bromberg-Martin et al., 2010; Hikosaka et al., 2014; Jin and Costa, 2010; Wilson, 2004; Yin and Knowlton, 2006). The striatum, the main input nucleus of the BG, receives afferents from diverse cortical areas—including the auditory cortex—and from numerous thalamic nuclei, including those of the non-lemniscal auditory thalamus (NLAT) in rodents (McGeorge and Faull, 1989; Hunnicutt et al., 2014, 2016; Oh et al., 2014). Recent studies combining functional imaging with viral-based anterograde tracing have offered new insights into the projection from the auditory cortex to the striatum (Nakata et al., 2020). In the canonical BG circuit (Wilson, 2004), the substantia nigra pars reticulata (SNr) and the internal segment of the globus pallidus (GPi) are the output nuclei, and the striatum projects directly to these nuclei in the direct pathway, and indirectly to these nuclei via the external segment of the globus pallidus (GPe) and the subthalamic nucleus (STN) in the indirect pathway; the BG output modulates thalamic nuclei that project to motor cortical areas. The posterior striatum, often referred to as the tail of the striatum (TS; a loosely defined structure discussed further in Section 3) is a major part of the striatum that receives auditory input (Chen et al., 2019; McGeorge and Faull, 1989; Miyamoto et al., 2018). Recent findings indicate that neurons in the TS, receiving input from the auditory cortex, project not to the SNr/GPi (cf. Aoki et al., 2019; Valjent and Gangarossa, 2021), but instead primarily to the posterior GPe, ultimately influencing the NLAT and the mesencephalic locomotor region (MLR) rather than the thalamic motor nucleus (Tomioka et al., 2024). In this review, we examine recent evidence regarding auditory inputs to the striatum and the resulting outputs from the TS in rodents. We then discuss the interaction between the BG and the auditory system and propose hypotheses on the functional significance of the auditory system–BG circuitry.

## 2 Auditory inputs to the striatum and other BG nuclei

In rodents, the striatum is divided into three functional regions: sensorimotor, associative, and limbic, which roughly correspond to the dorsolateral, dorsomedial, and ventral striatum, respectively (Yin and Knowlton, 2006; Thorn et al., 2010; Gruber and McDonald, 2012). A fourth region, the TS, has been identified based on the localization of corticostriatal and thalamostriatal inputs (Hunnicutt et al., 2016). Alternatively, the striatum can be compartmentalized into striosomes and matrix (Graybiel and Ragsdale, 1978). However, a narrow band along the dorsal and lateral margins, as well as part of the posterior regions of the striatum, is devoid of striosomes (Miyamoto et al., 2018).

The TS is, however, often vaguely defined. In rodents, the striatum has a large volume and comparable sizes along the mediolateral and dorsoventral dimensions in the rostral region, but tapers in volume and becomes dorsoventrally elongated toward the posterior end. An early rat study separated the striatum along the rostrocaudal direction into the body of the striatum and the TS, without a definition of border (Donoghue and Herkenham, 1986). Similarly, the TS is sometimes taken equal to the posterior striatum (Menegas et al., 2015; Pai and Monosov, 2022). A strict definition of the TS in rodents is “the extreme caudal part of the striatum” (Valjent and Gangarossa, 2021), whose ventral half exhibits a tri-laminar organization along the mediolateral axis, i.e., the medial division, the intermediate division, and the lateral division, with the intermediate division being striosome-free (Miyamoto et al., 2018, 2019). These three divisions can be characterized by the expression pattern of many marker molecules, including dopamine receptors (Gangarossa et al., 2013; Miyamoto et al., 2018, 2019; Ogata K. et al., 2022). We will use the term tTS to refer to the tri-laminar TS, and the term TS to refer to the posterior striatum including tTS.

**Figure 1:**
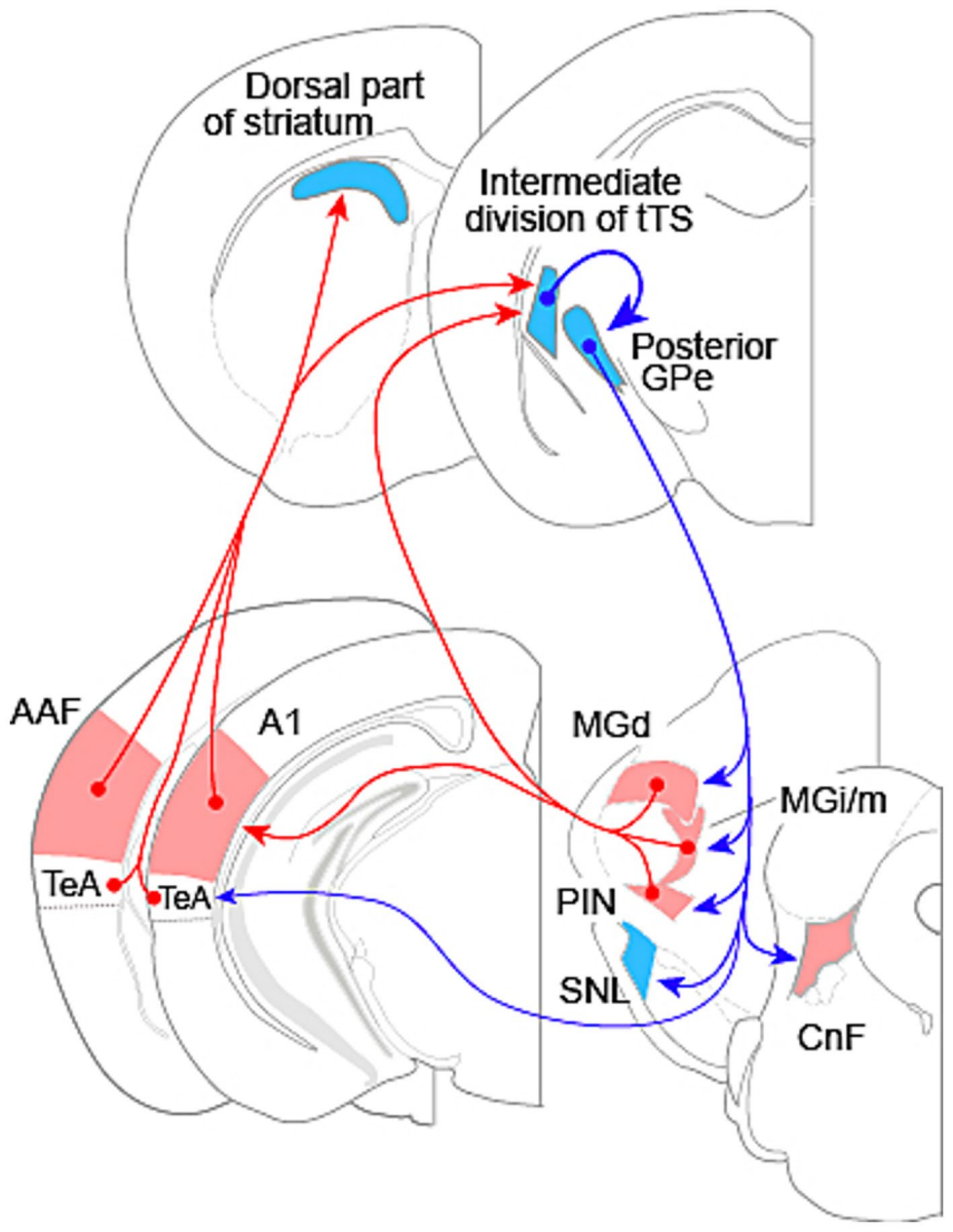
Interconnections between the auditory system and basal ganglia in rodents. Red arrows indicate excitatory connections; blue arrows indicate inhibitory connections. The ventral nucleus of the medial geniculate body is the primary drive for the auditory cortex, but is not illustrated here. The cortical area labeled as TeA also includes ventral auditory area and ectorhinal cortex. Abbreviations: AAF, anterior auditory field; A1, primary auditory cortex; CnF, cuneiform nucleus; GPe, external segment of the globus pallidus; MGd, dorsal nucleus of the medial geniculate body; MGi/m, internal and medial nuclei of the medial geniculate body; PIN, posterior intralaminar nucleus; SNL, substantia nigra lateralis; TeA, temporal association cortex. tTS: tri-laminar tail of striatum. Section diagrams are based on Paxinos and Franklin (2008).

Auditory input to the striatum was first shown for the auditory thalamus, the medial geniculate body (MGB). The MGB has a ventral subdivision (MGv), the largest component belonging to the ascending lemniscal auditory pathway, and several other smaller subdivisions, which are in the non-lemniscal pathway, including the dorsal nucleus (MGd), the medial nucleus (MGm), the internal nucleus (MGi), and the suprageniculate nucleus (Sun et al., 2025; Tomioka et al., 2023, 2024). Posterior thalamic nuclei adjacent to the MGB also participate in auditory processing, including the posterior intralaminar nucleus (PIN). In an early study, Ryugo and Killackey (1974) reported in rats that the MGm, but not the MGv, projects to the TS. Subsequent studies confirmed the projection in mice and rats (LeDoux et al., 1985; Ogata S. et al., 2022), and further localized the projection to the intermediate division of the tTS (Ogata S. et al., 2022). The medial region of MGB, however, may contain multiple subdivisions. Analysis of marker expression patterns in the MGB has identified the MGi, located between the MGv and the MGm (Tomioka et al., 2023). Previous injection sites may have included MGi, together with MGm. In addition, MGd and the PIN are also shown to project to the TS in a study using a retrograde viral tracer (Ponvert and Jaramillo, 2019) (Figure 1).

In an early rat study, McGeorge and Faull (1989) reported projections from the auditory cortex to the dorsal part of striatum and the TS. Two years later, LeDoux et al. (1991) further showed that the auditory cortex and the auditory thalamus projected to an overlapping region in the TS. Like the auditory cortex in primates (Kaas and Hackett, 2000), the auditory cortex in rodents has a core region and a surrounding belt region, with the core region comprising the primary auditory area (A1) and the anterior auditory field (AAF) and the belt region being consisted of several small fields (Nishimura et al., 2007; Polley et al., 2007; Sawatari et al., 2011; Stiebler et al., 1997). McGeorge and Faull (1989) appeared to have injected tracers into the entire auditory cortex, while LeDoux et al. (1991) restricted their injections to a cortical area ventral to Au1 (the core region according to brain atlas, which contains both A1 and AAF; Paxinos and Franklin, 2008), likely encompassing the ventral auditory area (AuV), temporal association area (TeA), and ectorhinal cortex. Subsequent studies traced projections from Au1 and have consistently found projections to the dorsal, striosome-free rostral striatum and the intermediate division of the tTS (Li et al., 2021; Miyamoto et al., 2018; Ogata S. et al., 2022; Tomioka et al., 2024; Xiong et al., 2015; Znamenskiy and Zador, 2013). Au1 projects to the dorsal region of the entire striatum other than the tTS, where the projection is confined to the intermediate division of tTS (Miyamoto et al., 2018). One study, however, has reported Au1 projection to a medial region of the caudal striatum, immediately adjacent to the GPe (Gangarossa et al., 2013). This discrepancy needs to be resolved in future studies.

Nakata et al. (2020) made injections of viral-based tracer into frequency-matched sites in functionally identified A1 and AAF, and found overlapping projections from these fields to both the dorsal part of striatum and the intermediate division of the tTS in mice. A1 and AAF receive parallel, independent inputs from the MGv (Takemoto et al., 2014). At this time, projections from the core auditory fields and AuV to the striatum is well established (Figure 1). It remains to be elucidated whether other auditory fields in the belt region project to the striatum in a similar way. Nevertheless, the projection from the core region and the AuV, together with the projections from the NLAT subdivisions to the tTS demonstrate that the striatum receives diverse auditory input from both the lemniscal and the non-lemniscal pathways.

The dual innervation of the striatum by cortical and thalamic auditory inputs raises the question whether the two inputs converge at the cellular level. Onto individual medium spiny neurons within the matrix compartment of the striatum, corticostriatal and thalamostriatal afferents have been shown anatomically to converge (Huerta-Ocampo et al., 2014). There is also electrophysiological evidence that cortical and thalamic projections converge onto individual striatal neurons in rat dorsal striatum (Smeal et al., 2008). Whether convergence occurs in the intermediate division of the tTS remains to be elucidated.

While there is consensus on the notion that the dorsal part of striatum integrates auditory, visual, and somatosensory inputs, two opposing views exist on the sensory inputs to the TS. One view is that the TS receives auditory input exclusively (Chen et al., 2019; McGeorge and Faull, 1989; Miyamoto et al., 2018), while the other posits convergence of auditory, visual, and somatosensory inputs (Lee et al., 2023; Hunnicutt et al., 2016; Oh et al., 2014; Valjent and Gangarossa, 2021). This discrepancy may stem from studies focusing on different striatal regions: the former on the tTS, and the latter on the posterior striatum anterior to the tTS. Miyamoto et al. (2018) clearly demonstrated that the posterior striatum rostral to the tTS receives cortical inputs in a manner similar to rostral striatum: auditory, visual, and somatosensory inputs to the dorsal region and motor input to the lateral region, and that the tTS receives input only from the auditory cortex at its intermediate division. The term auditory striatum has been used in prior literature (Chen et al., 2019; Nakajima et al., 2019); here, we propose defining it as the intermediate division of the tTS.

The striatum receives auditory input not only directly from the auditory cortex and auditory thalamus but also indirectly from nuclei outside the canonical auditory pathway. The superior colliculus (SC) relays auditory information from the inferior colliculus (Mellott et al., 2018) and from the auditory cortex (Nakata et al., 2020; Benavidez et al., 2021) to the dorsal striatum via the parafascicular nucleus of the thalamus (Melleu and Canteras, 2024).

In addition to the striatum, several other nuclei within the basal ganglia also receive auditory inputs. Dopamine neurons in the substantia nigra pars compacta have long been known to respond to sound stimulation (Steinfels et al., 1983), and a subset of STN neurons responds to auditory stimuli with short latency (Mirzaei et al., 2017). These auditory responses in the substantia nigra and STN may originate from inputs conveyed through the SC (Al Tannir et al., 2022; Melleu and Canteras, 2024).

## 3 Output of the BG to the auditory system and other brain regions

Neuronal tracer studies have demonstrated projections from the rodent TS to the GPe (Li et al., 2021; Tulloch et al., 1978). Recently, using a combination of viral vectors in transgenic mice for cell-type-specific trans-synaptic tracing, Tomioka et al. (2024) demonstrated that GABAergic neurons in the tTS, which receive input from the Au1, project primarily to the posterior GPe (Figure 1), with a minor projection to the substantia nigra lateralis (SNL). Tracer injections into the intermediate division of the tTS also result in labeling of the posterior GPe and the SNL (Ogata K. et al., 2022). The majority of cells in the intermediate division of tTS, or the auditory striatum, expresses dopamine receptor D2 (Miyamoto et al., 2019; Ogata K. et al., 2022); the projection from this division to GPe is thus consistent with the indirect pathway in canonical BG circuit (Ogata K. et al., 2022; Valjent and Gangarossa, 2021).

GABAergic neurons in the posterior GPe innervate several target regions in the thalamus, brainstem, and temporal cortex (Figure 1). Among these targets, the subdivisions of the NLAT and the PIN receive the strongest input; the SNL and the cuneiform nucleus (CnF) receive moderate input; and the TeA receives the weakest input (Tomioka et al., 2024; Figure 1). The STN—the target of GPe in the canonical BG circuit (Hikosaka et al., 2014; Wilson, 2004)—receives minimal input from the posterior GPe (Tomioka et al., 2024). Projections from the GPe receiving input from the auditory striatum to the auditory sector of the thalamic reticular nucleus have been reported (Nakajima et al., 2019); however, this pathway was not consistently observed in the study by Tomioka et al. (2024). This discrepancy warrants clarification in future investigations.

Additional tTS-related outputs are mediated by large GABAergic neurons in the medial division of the tTS, whose dendrites extend into the intermediate division and receive input from both the auditory cortex and auditory thalamus. These neurons project to the zona incerta and the ventral medial nucleus of the thalamus (Ogata S. et al., 2022).

## 4 Functional interactions of the auditory system and the BG

Inputs from the auditory cortex and thalamus drive robust sound responses in neurons of the TS (Bordi and LeDoux, 1992; Bordi et al., 1993; Guo et al., 2018). Recent studies have demonstrated distinct roles for cortical and thalamic inputs in producing the responses of TS neurons to sound. Ponvert and Jaramillo (2019) examined the auditory responses of identified cortical neurons and thalamic neurons that project to the TS, and found that both the cortical neurons and the thalamic neurons respond to a broad range of tone frequencies and broadband noise, with thalamic neurons capable of following higher amplitude modulation frequencies. This latter finding aligns with the general trend that the highest amplitude modulation frequency a neuron can follow gradually decreases along the ascending auditory pathway (Joris et al., 2004). Thalamic inputs may therefore convey more precise temporal information to the TS than cortical inputs. In the frequency domain, Chen et al. (2019) demonstrated that thalamic input controls the response gain of TS neurons to auditory stimuli, while cortical input provides frequency tuning information to TS neurons. In rodents, neurons in the AAF are more broadly tuned to frequency (Hackett, 2011; Guo et al., 2012), and can follow faster temporal modulations than A1 neurons (Polley et al., 2007; Linden et al., 2003; Sołyga and Barkat, 2019). Although axon terminals from both A1 and AAF overlap in tTS (Nakata et al., 2020), it remains to be elucidated whether A1 and AAF inputs converge onto the same tTS neuron, and how their different response properties manifest in the auditory response of striatal neurons.

**Figure 2:**
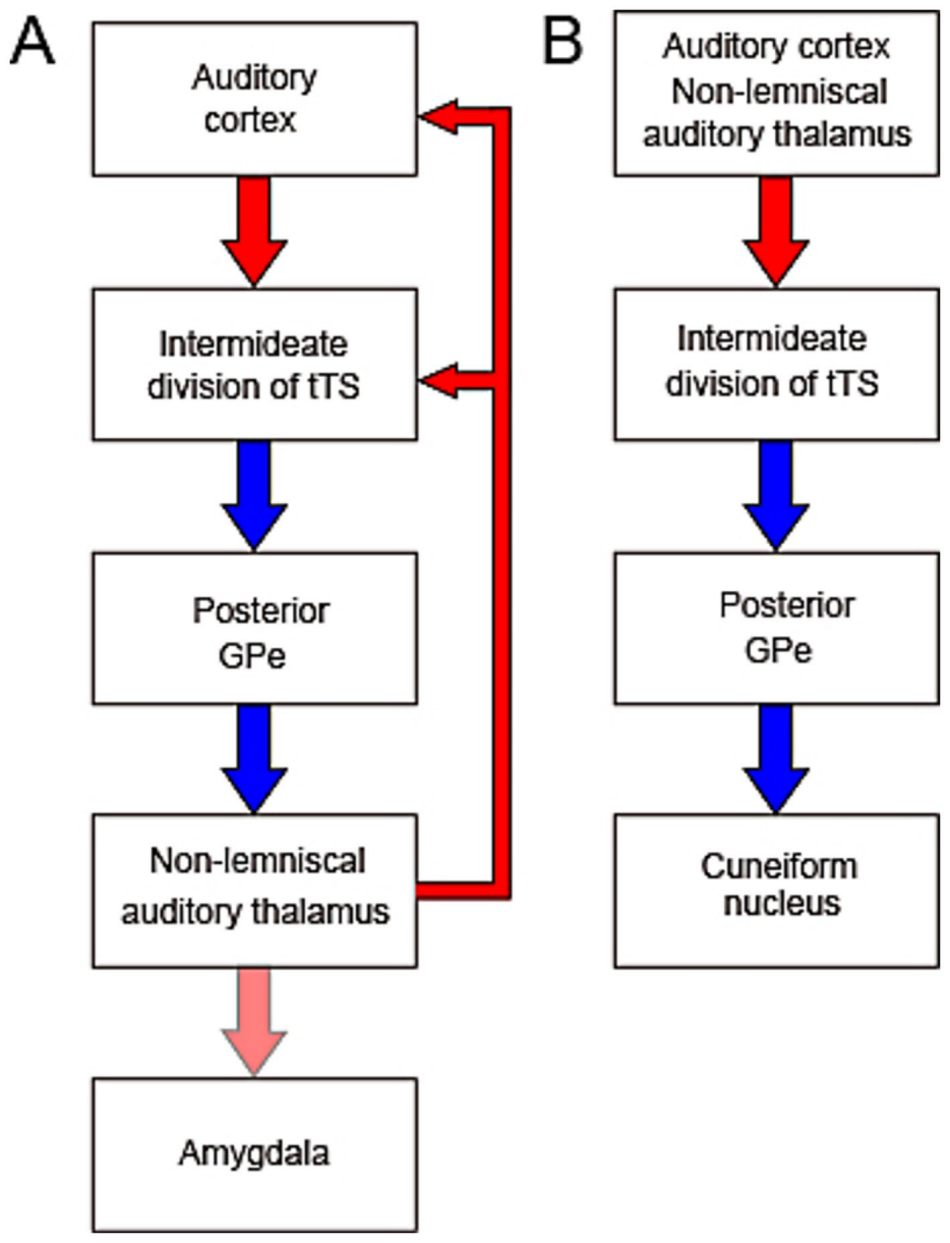
Proposed feedback **(A)** and feedforward **(B)** circuits illustrating functional hypotheses for the auditory system–basal ganglia interaction. **(A)** The intermediate division of tri-laminar tail of the striatum (tTS) receives auditory input from both the auditory cortex and the non-lemniscal auditory thalamus (NLAT). Through inhibition of the posterior GPe, which is itself inhibitory, the tTS may disinhibit NLAT output and thus modulates auditory cortical activity. The NLAT and its downstream target, the amygdala, also receive additional inputs and are involved in diverse auditory-related functions. **(B)** The feedforward circuit from the auditory cortex and thalamus to the cuneiform nucleus (CnF) via the basal ganglia may mediate sound-induced activation of the CnF through disinhibition. Given the CnF’s role as part of the mesencephalic locomotor region, this circuit may underlie movement initiation and modulation of locomotor speed in response to auditory stimuli.

What functional roles might auditory inputs to the TS play? One function of the corticostriatal pathway is to drive decision making in sound-cued multichoice behavior (Xiong et al., 2015; Znamenskiy and Zador, 2013). Dopaminergic input to the TS is virtually exclusively from the SNL (Menegas et al., 2015), which may carry reinforcement signal modifying synaptic efficacy in the auditory corticostriatal and/or thalamostriatal pathways, and thereby implement the formation of behavioral choice. The auditory corticostriatal pathway has also been shown to mediate sound-induced defense behaviors (Li et al., 2021). The pathway from the auditory cortex and thalamus to the CnF reported by Tomioka et al. (2024), constitutes a disinhibitory pathway (Figure 2B), in which CnF is excited by the cortex and thalamus via disinhibition. This discovery suggests that the CnF may serve as a downstream component to the corticostriatal pathway in mediating escape behavior, as the CnF is interconnected with the periaqueductal gray and participates in mediating defensive behavior (Bindi et al., 2023). It remains to be investigated how the sound response properties of striatal neurons (Chen et al., 2019), shaped by cortical and thalamic inputs (Ponvert and Jaramillo, 2019), relate to their functional roles.

The recent discovery of projections from GABAergic neurons in the posterior GPe to the NLAT subdivisions and the PIN (Tomioka et al., 2024) raises the possibility that the auditory system and BG form a loop circuit, in which NLAT neurons may be excited by cortical neurons via disinhibition mediated by the BG circuitry (Figure 1; Figure 2A). How the BG influence the activity of the NLAT neurons remains unknown. Because GPe neurons typically exhibit persistent and continuous firing at a high rate (Bevan et al., 2002), the BG may suppress NLAT activity in the absence of auditory input to the auditory striatum. Considering that A1 and AAF are primarily driven by the lemniscal MGB, i.e., MGv, it is tempting to hypothesize that the lemniscal auditory pathway (from MGv to A1 and AAF) may gate NLAT neuron activity dynamically via disinhibition through the BG loop (Figure 2A). In turn, the NLAT, under BG control, modulates auditory cortical activity. Because the tTS receives only auditory input (Chen et al., 2019; Miyamoto et al., 2018), the auditory system-BG loop might not be a substrate mediating movement-related modulation of auditory cortical responses, a modulation effect that is now well documented (Morandell et al., 2024).

All subdivisions of the NLAT, along with the PIN, are known to project to the amygdala (LeDoux et al., 1991; Figure 2A). The NLAT, its calretinin-expressing neurons in particular, plays a critical role in sound-cued aversive associative learning (Barsy et al., 2020; Weinberger, 2011). The BG may therefore modulate emotional responses and sound-cued learning via these pathways.

## 5 Control of the CnF by the auditory system and BG

The posterior GPe serves as the output nucleus of the BG in the recently identified auditory system-BG loop (Tomioka et al., 2024; Figures 1, 2), in contrast to the canonical BG circuit, where the GPi and SNr act as output nuclei (Hikosaka et al., 2014; Wilson, 2004). One major target of the posterior GPe, deviating away from the loop circuit, is the CnF (Figures 1, 2B), which, along with the pedunculopontine nucleus, constitutes the MLR (Noga and Whelan, 2022; Ryczko, 2024; cf. Bindi et al., 2023). The CnF contains both excitatory glutamatergic neurons and inhibitory GABAergic interneurons (Ryczko, 2024), and activation of glutamatergic neurons can initiate locomotion and regulate locomotor speed (Caggiano et al., 2018; Josset et al., 2018). Behaviorally, the CnF is involved not only in escape behavior, but also in normal walking (Noga and Whelan, 2022; cf. Bindi et al., 2023). Because activation of the GABAergic neuron can have opposing effects on locomotion (Ryczko, 2024), the exact function of the posterior GPe input to CnF depends on the postsynaptic cell type. It is tempting to speculate that the auditory cortex and thalamus may enhance CnF activity via disinhibition of GPe neurons, leading to the initiation or modulation of locomotion (Figure 2B). This hypothesis might relate to the human ability to dance in response to music. The therapeutic effects of rhythmic auditory stimulation on gait in patients with Parkinson’s disease, in which the MLR shows decreased activity (Ryczko and Dubuc, 2017), are consistent with this hypothesis. In this regard, the intermediate division of the tTS has low level of tyrosine hydroxylase (Miyamoto et al., 2019), suggesting that dopaminergic modulation may be reduced in this region. Therefore, the pathway from the auditory cortex and thalamus to the CnF via the tTS (see Figure 2B) may remain relatively preserved in Parkinson’s disease, potentially supporting sound-guided movement. However, caution is warranted when extending the discussion of the rodent auditory system-BG-CnF circuit to humans, since the TS in primates may receive cortical input primarily from the ventral inferior temporal cortex, a region primarily associated with visual processing (Lee et al., 2023). However, the presence of input from the NLAT to the TS in primates supports the relevance of this hypothesis for investigation in primates.

The feedforward circuit from the auditory system to the CnF via the BG predicts that movement is reactive to sound stimuli. Any neural circuit linking the auditory system to motor centers should also predict reactive movement. While this is consistent with a rat study showing reactive movement to sound of regular rhythm (Katsu et al., 2021), a recent study, however, shows evidence of predictive motor behavior in both rats and humans (Ito et al., 2022). Some form of adaptive mechanism must be invoked to account for predictive movement.

The circuit show in Figure 2B is by no means the only circuit linking the auditory system to motor-related brain areas. The output of the dorsal part of striatum which also receives auditory input (Miyamoto et al., 2018; Nakata et al., 2020), remains to be explored, and might be linked to motor related thalamic nuclei. The secondary motor cortex (Nakata et al., 2020), cerebellum (Wolfe, 1972), and other subcortical motor-related structures (Xiao et al., 2023) are also linked with the auditory system.

## 6 Future directions

While the influence of auditory cortical and thalamic inputs on the sound responses of TS neurons is now well characterized, how BG innervation of the NLAT modulates auditory cortical responses remains to be elucidated. In this review, we proposed the hypothesis that the lemniscal auditory pathway regulates NLAT activity via BG-mediated disinhibition (Figure 2A). This hypothesis awaits experimental validation. We also hypothesize that the CnF, regulated by the auditory system via the BG, mediates sound-driven modulation of movement (Figure 2B)—a concept that likewise requires functional validation. To better understand how the auditory system–BG loop functions, the circuit diagram shown in Figure 1 must be refined to a cell-level resolution.

## References

[ref1] Al Tannir R. Pautrat A. Baufreton J. Overton P. Coizet V. (2022). The subthalamic nucleus: a hub for sensory control via short threelateral loop connections with the brainstem? Curr. Neuropharmacol. 21, 22–30. doi: 10.2174/1570159X20666220718113548 PMC10193764 35850655

[ref2] Aoki S. Smith J. B. Li H. Yan X. Igarashi M. Coulon P.. (2019). An open cortico-basal ganglia loop allows limbic control over motor output via the nigrothalamic pathway. eLife 8:e49995. doi: 10.7554/eLife.49995, PMID: 31490123 PMC6731092

[ref9001] Barsy B. Kocsis K. Magyar A. Babiczky Á. Szabó M. Veres J. M. (2020). Associative and plastic thalamic signaling to the lateral amygdala controls fear behavior. Nat. Neurosci. 23, 625–637. doi: 10.1038/s41593-020-0620-z, PMID: 32284608

[ref3] Benavidez N. L. Bienkowski M. S. Zhu M. Garcia L. H. Fayzullina M. Gao L.. (2021). Organization of the inputs and outputs of the mouse superior colliculus. Nat. Commun. 12:4004. doi: 10.1038/s41467-021-24241-2, PMID: 34183678 PMC8239028

[ref4] Bevan M. D. Magill P. J. Terman D. Bolam J. P. Wilson C. J. (2002). Move to the rhythm: oscillations in the subthalamic nucleus–external globus pallidus network. Trends Neurosci. 25, 525–531. doi: 10.1016/s0166-2236(02)02235-x, PMID: 12220881

[ref5] Bindi R. P. Guimarães C. C. de Oliveira A. R. Melleu F. F. de Lima M. A. X. Baldo M. V. C.. (2023). Anatomical and functional study of the cuneiform nucleus: a critical site to organize innate defensive behaviors. Ann. N. Y. Acad. Sci. 1521, 79–95. doi: 10.1111/nyas.14954, PMID: 36606723

[ref6] Bordi F. LeDoux J. (1992). Sensory tuning beyond the sensory system: an initial analysis of auditory response properties of neurons in the lateral amygdaloid nucleus and overlying areas of the striatum. J. Neurosci. 12, 2493–2503. doi: 10.1523/JNEUROSCI.12-07-02493.1992, PMID: 1613543 PMC6575825

[ref7] Bordi F. LeDoux J. Clugnet M. C. Pavlides C. (1993). Single-unit activity in the lateral nucleus of the amygdala and overlying areas of the striatum in freely behaving rats: rates, discharge patterns, and responses to acoustic stimuli. Behav. Neurosci. 107, 757–769. doi: 10.1037/0735-7044.107.5.757, PMID: 8280386

[ref8] Bromberg-Martin E. S. Matsumoto M. Hikosaka O. (2010). Dopamine in motivational control: rewarding, aversive, and alerting. Neuron 68, 815–834. doi: 10.1016/j.neuron.2010.11.022, PMID: 21144997 PMC3032992

[ref9] Caggiano V. Leiras R. Goñi-Erro H. Masini D. Bellardita C. Bouvier J.. (2018). Midbrain circuits that set locomotor speed and gait selection. Nature 553, 455–460. doi: 10.1038/nature25448, PMID: 29342142 PMC5937258

[ref10] Chen L. Wang X. Ge S. Xiong Q. (2019). Medial geniculate body and primary auditory cortex differentially contribute to striatal sound representations. Nat. Commun. 10:418. doi: 10.1038/s41467-019-08350-7, PMID: 30679433 PMC6346050

[ref11] Donoghue J. P. Herkenham M. (1986). Neostriatal projections from individual cortical fields conform to histochemically distinct striatal compartments in the rat. Brain Res. 365, 397–403. doi: 10.1016/0006-8993(86)91658-6, PMID: 3004664

[ref12] Gangarossa G. Espallergues J. Mailly P. De Bundel D. de Kerchove d.’ E. A. Hervé D.. (2013). Spatial distribution of D1R- and D2R-expressing medium-sized spiny neurons differs along the rostro-caudal axis of the mouse dorsal striatum. Front. Neural Circuits 7:124. doi: 10.3389/fncir.2013.00124 23908605 PMC3725430

[ref13] Graybiel A. M. Ragsdale C. W. (1978). Histochemically distinct compartments in the striatum of human, monkey, and cat demonstrated by acetylthiocholinesterase staining. Proc. Natl. Acad. Sci. USA 75, 5723–5725. doi: 10.1073/pnas.75.11.5723 103101 PMC393041

[ref14] Gruber A. J. McDonald R. J. (2012). Context, emotion, and the strategic pursuit of goals: interactions among multiple brain systems controlling motivated behavior. Front. Behav. Neurosci. 6:50. doi: 10.3389/fnbeh.2012.00050 22876225 PMC3411069

[ref15] Guo W. Chambers A. R. Darrow K. N. Hancock K. E. Shinn-Cunningham B. G. Polley D. B. (2012). Robustness of cortical topography across fields, laminae, anesthetic states, and neurophysiological signal types. J. Neurosci. 32, 9159–9172. doi: 10.1523/JNEUROSCI.0065-12.2012, PMID: 22764225 PMC3402176

[ref16] Guo L. Walker W. I. Ponvert N. D. Penix P. L. Jaramillo S. (2018). Stable representation of sounds in the posterior striatum during flexible auditory decisions. Nat. Commun. 9:1534. doi: 10.1038/s41467-018-03994-3, PMID: 29670112 PMC5906458

[ref17] Hackett T. A. (2011). Information flow in the auditory cortical network. Hear. Res. 271, 133–146. doi: 10.1016/j.heares.2010.01.011, PMID: 20116421 PMC3022347

[ref18] Hikosaka O. Kim H. K. Yasuda M. Yamamoto S. (2014). BG circuits for reward value-guided behavior. Annu. Rev. Neurosci. 37, 289–306. doi: 10.1146/annurev-neuro-071013-013924, PMID: 25032497 PMC4148825

[ref19] Huerta-Ocampo I. Mena-Segovia J. Bolam J. P. (2014). Convergence of cortical and thalamic input to direct and indirect pathway medium spiny neurons in the striatum. Brain Struct. Funct. 219, 1787–1800. doi: 10.1007/s00429-013-0601-z, PMID: 23832596 PMC4147250

[ref20] Hunnicutt B. J. Jongbloets B. C. Birdsong W. T. Gertz K. J. Zhong H. Mao T. (2016). A comprehensive excitatory input map of the striatum reveals novel functional organization. eLife 5:e19103. doi: 10.7554/eLife.19103, PMID: 27892854 PMC5207773

[ref21] Hunnicutt B. J. Long B. R. Kusefoglu D. Gertz K. J. Zhong H. Mao T. (2014). A comprehensive thalamocortical projection map at the mesoscopic level. Nat. Neurosci. 17, 1276–1285. doi: 10.1038/nn.3780, PMID: 25086607 PMC4152774

[ref22] Ito Y. Shiramatsu T. I. Ishida N. Oshima K. Magami K. Takahashi H. (2022). Spontaneous beat synchronization in rats: neural dynamics and motor entrainment. Sci. Adv. 8:eabo7019. doi: 10.1126/sciadv.abo7019, PMID: 36367945 PMC9651867

[ref23] Jin X. Costa R. M. (2010). Start/stop signals emerge in nigrostriatal circuits during sequence learning. Nature 466, 457–462. doi: 10.1038/nature09263, PMID: 20651684 PMC3477867

[ref24] Joris P. X. Schreiner C. E. Rees A. (2004). Neural processing of amplitude-modulated sounds. Physiol. Rev. 84, 541–577. doi: 10.1152/physrev.00029.2003, PMID: 15044682

[ref25] Josset N. Roussel M. Lemieux M. Lafrance-Zoubga D. Rastqar A. Bretzner F. (2018). Distinct contributions of mesencephalic locomotor region nuclei to locomotor control in the freely behaving mouse. Curr. Biol. 28, 884–901.e3. doi: 10.1016/j.cub.2018.02.007, PMID: 29526593

[ref26] Kaas J. H. Hackett T. A. (2000). Subdivisions of auditory cortex and processing streams in primates. Proc. Natl. Acad. Sci. USA 97, 11793–11799. doi: 10.1073/pnas.97.22.11793, PMID: 11050211 PMC34351

[ref27] Katsu N. Yuki S. Okanoya K. (2021). Production of regular rhythm induced by external stimuli in rats. Anim. Cogn. 24, 1133–1141. doi: 10.1007/s10071-021-01505-4, PMID: 33751275

[ref28] LeDoux J. E. Farb C. R. Romanski L. M. (1991). Overlapping projections to the amygdala and striatum from auditory processing areas of the thalamus and cortex. Neurosci. Lett. 134, 139–144. doi: 10.1016/0304-3940(91)90526-Y, PMID: 1815147

[ref29] LeDoux J. E. Ruggiero D. A. Reis D. J. (1985). Projections to the subcortical forebrain from anatomically defined regions of the medial geniculate body in the rat. J. Comp. Neurol. 242, 182–213. doi: 10.1002/cne.902420204, PMID: 4086664

[ref30] Lee K. An S. Y. Park J. Lee S. Kim H. F. (2023). Anatomical and functional comparison of the caudate tail in primates and the tail of the striatum in rodents: implications for sensory information processing and habitual behavior. Mol. Cells 46, 461–469. doi: 10.14348/molcells.2023.0051, PMID: 37455248 PMC10440267

[ref31] Li Z. Wei J. X. Zhang G. W. Huang J. J. Zingg B. Wang X.. (2021). Corticostriatal control of defense behavior in mice induced by auditory looming cues. Nat. Commun. 12:1040. doi: 10.1038/s41467-021-21248-7, PMID: 33589613 PMC7884702

[ref32] Linden J. F. Liu R. C. Sahani M. Schreiner C. E. Merzenich M. M. (2003). Spectrotemporal structure of receptive fields in areas AI and AAF of mouse auditory cortex. J. Neurophysiol. 90, 2660–2675. doi: 10.1152/jn.00751.2002, PMID: 12815016

[ref33] McGeorge A. J. Faull R. L. (1989). The organization of the projection from the cerebral cortex to the striatum in the rat. Neuroscience 29, 503–537. doi: 10.1016/0306-4522(89)90128-0 2472578

[ref34] Melleu F. F. Canteras N. S. (2024). Pathways from the superior colliculus to the basal ganglia. Curr. Neuropharmacol. 22, 1431–1453. doi: 10.2174/1570159X21666230911102118, PMID: 37702174 PMC11097988

[ref35] Mellott J. G. Beebe N. L. Schofield B. R. (2018). GABAergic and non-GABAergic projections to the superior colliculus from the auditory brainstem. Brain Struct. Funct. 223, 1923–1936. doi: 10.1007/s00429-017-1599-4, PMID: 29302743 PMC5886796

[ref36] Menegas W. Bergan J. F. Ogawa S. K. Isogai Y. Venkataraju K. U. Osten P.. (2015). Dopamine neurons projecting to the posterior striatum form an anatomically distinct subclass. eLife 4:e1003. doi: 10.7554/eLife.10032, PMID: 26322384 PMC4598831

[ref37] Mirzaei A. Kumar A. Leventhal D. Mallet N. Aertsen A. Berke J.. (2017). Sensorimotor processing in the basal ganglia leads to transient beta oscillations during behavior. J. Neurosci. 37, 11220–11232. doi: 10.1523/JNEUROSCI.1289-17.2017, PMID: 29038241 PMC6596813

[ref38] Miyamoto Y. Katayama S. Shigematsu N. Nishi A. Fukuda T. (2018). Striosome-based map of the mouse striatum that is conformable to both cortical afferent topography and uneven distributions of dopamine D1 and D2 receptor-expressing cells. Brain Struct. Funct. 223, 4275–4291. doi: 10.1007/s00429-018-1749-3, PMID: 30203304 PMC6267261

[ref39] Miyamoto Y. Nagayoshi I. Nishi A. Fukuda T. (2019). Three divisions of the mouse caudal striatum differ in the proportions of dopamine D1 and D2 receptor-expressing cells, distribution of dopaminergic axons, and composition of cholinergic and GABAergic interneurons. Brain Struct. Funct. 224, 2703–2716. doi: 10.1007/s00429-019-01928-3, PMID: 31375982 PMC6778543

[ref40] Morandell K. Yin A. Del Rio R. T. Schneider D. M. (2024). Movement-related modulation in mouse auditory cortex is widespread yet locally diverse. J. Neurosci. 44:e1227232024. doi: 10.1523/JNEUROSCI.1227-23.2024 38286628 PMC10941236

[ref41] Nakajima M. Schmitt L. I. Halassa M. M. (2019). Prefrontal cortex regulates sensory filtering through a basal ganglia-to-thalamus pathway. Neuron 103, 445–458.e10. doi: 10.1016/j.neuron.2019.05.026, PMID: 31202541 PMC6886709

[ref42] Nakata S. Takemoto M. Song W.-J. (2020). Differential cortical and subcortical projection targets of subfields in the core region of mouse auditory cortex. Hear. Res. 386:107876. doi: 10.1016/j.heares.2019.107876, PMID: 31881516

[ref43] Nishimura M. Shirasawa H. Kaizo H. Song W.-J. (2007). New field with tonotopic organization in Guinea pig auditory cortex. J. Neurophysiol. 97, 927–932. doi: 10.1152/jn.00689.2006 17050828

[ref44] Noga B. R. Whelan P. J. (2022). The mesencephalic locomotor region: beyond locomotor control. Front. Neural Circuits 16:884785. doi: 10.3389/fncir.2022.884785, PMID: 35615623 PMC9124768

[ref45] Ogata K. Kadono F. Hirai Y. Inoue K. Takada M. Karube F.. (2022). Conservation of the direct and indirect pathway dichotomy in mouse caudal striatum with uneven distribution of dopamine receptor D1- and D2-expressing neurons. Front. Neuroanat. 16:809446. doi: 10.3389/fnana.2022.809446, PMID: 35185482 PMC8854186

[ref46] Ogata S. Miyamoto Y. Shigematsu N. Esumi S. Fukuda T. (2022). The tail of the mouse striatum contains a novel large type of GABAergic neuron incorporated in a unique disinhibitory pathway that relays auditory signals to subcortical nuclei. J. Neurosci. 42, 8078–8094. doi: 10.1523/JNEUROSCI.2236-21.2022, PMID: 36104279 PMC9637004

[ref47] Oh S. W. Harris J. A. Ng L. Winslow B. Cain N. Mihalas S.. (2014). A mesoscale connectome of the mouse brain. Nature 508, 207–214. doi: 10.1038/nature13186, PMID: 24695228 PMC5102064

[ref48] Pai J. Monosov I. E. (2022). Dopamine in the rodent tail of striatum regulates behavioral variability in response to threatening novel objects. Neuron 110, 3653–3655. doi: 10.1016/j.neuron.2022.10.019, PMID: 36395752

[ref49] Paxinos G. Franklin K. B. J. (2008). The mouse brain in stereotaxic coordinates, compact. Cambridge, Massachusetts: Elsevier Academic Press.

[ref50] Polley D. B. Read H. L. Storace D. A. Merzenich M. M. (2007). Multiparametric auditory receptive field organization across five cortical fields in the albino rat. J. Neurophysiol. 97, 3621–3638. doi: 10.1152/jn.01298.2006, PMID: 17376842

[ref51] Ponvert N. D. Jaramillo S. (2019). Auditory thalamostriatal and corticostriatal pathways convey complementary information about sound features. J. Neurosci. 39, 271–280. doi: 10.1523/JNEUROSCI.1188-18.2018, PMID: 30459227 PMC6325256

[ref52] Ryczko D. (2024). The mesencephalic locomotor region: multiple cell types, multiple behavioral roles, and multiple implications for disease. Neuroscientist 30, 347–366. doi: 10.1177/10738584221139136, PMID: 36575956 PMC11107129

[ref53] Ryczko D. Dubuc R. (2017). Dopamine and the brainstem locomotor networks: from lamprey to human. Front. Neurosci. 11:295. doi: 10.3389/fnins.2017.00295, PMID: 28603482 PMC5445171

[ref54] Ryugo D. K. Killackey H. P. (1974). Differential telencephalic projections of the medial and ventral divisions of the medial geniculate body of the rat. Brain Res. 82, 173–177. doi: 10.1016/0006-8993(74)90903-2, PMID: 4611594

[ref55] Sawatari H. Tanaka Y. Takemoto M. Nishimura M. Hasegawa K. Saitoh K.. (2011). Identification and characterization of an insular auditory field in mice. Eur. J. Neurosci. 34, 1944–1952. doi: 10.1111/j.1460-9568.2011.07926.x, PMID: 22118307

[ref56] Smeal R. M. Keefe K. A. Wilcox K. S. (2008). Differences in excitatory transmission between thalamic and cortical afferents to single spiny efferent neurons of rat dorsal striatum. Eur. J. Neurosci. 28, 2041–2052. doi: 10.1111/j.1460-9568.2008.06505.x, PMID: 19046385 PMC2596669

[ref57] Sołyga M. Barkat T. R. (2019). Distinct processing of tone offset in two primary auditory cortices. Sci. Rep. 9:9581. doi: 10.1038/s41598-019-45952-z, PMID: 31270350 PMC6610078

[ref58] Steinfels G. F. Heym J. Strecker R. E. Jacobs B. L. (1983). Response of dopaminergic neurons in cat to auditory stimuli presented across the sleep–waking cycle. Brain Res. 277, 150–154. doi: 10.1016/0006-8993(83)90917-4, PMID: 6640288

[ref59] Stiebler I. Neulist R. Fichtel I. Ehret G. (1997). The auditory cortex of the house mouse: left-right differences, tonotopic organization and quantitative analysis of frequency representation. J. Comp. Physiol. A 181, 559–571. doi: 10.1007/s003590050140, PMID: 9449817

[ref60] Sun M. Takemoto M. Tomioka R. Dong C. Lin A. Song W.-J. (2025). Axon terminal distribution in layer 1 of the mouse auditory cortex: differential projections from the dorsal and medial subdivisions of the medial geniculate body and the marginal zone of the posterior thalamic nuclei. Hear. Res. 462:109275. doi: 10.1016/j.heares.2025.109275, PMID: 40279885

[ref61] Takemoto M. Hasegawa K. Nishimura M. Song W.-J. (2014). The insular auditory field receives input from the lemniscal subdivision of the auditory thalamus in mice. J. Comp. Neurol. 522, 1373–1389. doi: 10.1002/cne.23491, PMID: 24638871

[ref62] Thorn C. A. Atallah H. Howe M. Graybiel A. M. (2010). Differential dynamics of activity changes in dorsolateral and dorsomedial striatal loops during learning. Neuron 66, 781–795. doi: 10.1016/j.neuron.2010.04.036, PMID: 20547134 PMC3108575

[ref63] Tomioka R. Shigematsu N. Miyashita T. Takahashi Y. Yamamoto M. Yoshimura Y.. (2024). The external globus pallidus as the hub of the auditory cortico-BG loop. eNeuro 11:ENEURO.0161-24.2024. doi: 10.1523/ENEURO.0161-24.2024 PMC11594937 39592219

[ref64] Tomioka R. Takemoto M. Song W.-J. (2023). Neurochemical properties for defining subdivisions of the medial geniculate body. Hear. Res. 431:108724. doi: 10.1016/j.heares.2023.108724 36871497

[ref65] Tulloch I. F. Arbuthnott G. W. Wright A. K. (1978). Topographical organization of the striatonigral pathway revealed by anterograde and retrograde neuroanatomical tracing techniques. J. Anat. 127, 425–441, PMID: 721701 PMC1235782

[ref66] Valjent E. Gangarossa G. (2021). The tail of the striatum: from anatomy to connectivity and function. Trends Neurosci. 44, 203–214. doi: 10.1016/j.tins.2020.10.016, PMID: 33243489

[ref67] Weinberger N. M. (2011). The medial geniculate, not the amygdala, as the root of auditory fear conditioning. Hear. Res. 274, 61–74. doi: 10.1016/j.heares.2010.03.093, PMID: 20466051 PMC2949681

[ref68] Wilson C. J. (2004). “Basal ganglia” in The synaptic Organization of the Brain. ed. Shepherd G. M. (New York: Oxford University Press), 361–413.

[ref69] Wolfe J. W. (1972). Responses of the cerebellar auditory area to pure tone stimuli. Exp. Neurol. 36, 295–309. doi: 10.1016/0014-4886(72)90025-8, PMID: 5053356

[ref70] Xiao C. Wei J. Zhang G.-W. Tao C. Huang J. J. Shen L.. (2023). Glutamatergic and GABAergic neurons in pontine central gray mediate opposing valence-specific behaviors through a global network. Neuron 111, 1486–1503.e7. doi: 10.1016/j.neuron.2023.02.012, PMID: 36893756 PMC10164086

[ref71] Xiong Q. Znamenskiy P. Zador A. M. (2015). Selective corticostriatal plasticity during acquisition of an auditory discrimination task. Nature 521, 348–351. doi: 10.1038/nature14225, PMID: 25731173 PMC4454418

[ref72] Yin H. H. Knowlton B. J. (2006). The role of the basal ganglia in habit formation. Nat. Rev. Neurosci. 7, 464–476. doi: 10.1038/nrn1919 16715055

[ref73] Znamenskiy P. Zador A. M. (2013). Corticostriatal neurons in auditory cortex drive decisions during auditory discrimination. Nature 497, 482–485. doi: 10.1038/nature12077, PMID: 23636333 PMC3670751

